# Predictors of AIDS-related death among adult HIV-infected inpatients in Kisangani, the Democratic Republic of Congo

**DOI:** 10.11604/pamj.2020.37.144.25802

**Published:** 2020-10-08

**Authors:** Alliance Tagoto Tepungipame, Serge Tonen-Wolyec, Ginette Claude Kalla, Eugeune Basandja Longembe, Rachel Olonga Atike, Joris Losimba Likwela, Francois-Xavier Mbopi-Kéou, Laurent Bélec, Salomon Batina-Agasa

**Affiliations:** 1 Faculty of Medicine and Pharmacy, University of Kisangani, Kisangani, Democratic Republic of the Congo,; 2 National AIDS and STIs Control Programme, Kisangani, Democratic Republic of the Congo,; 3 Ecole Doctorale Régionale d´Afrique Centrale en Infectiologie Tropicale, Franceville, Gabon,; 4 Faculty of Medicine and Biomedical Sciences, University of Yaoundé I, Yaoundé, Cameroon,; 5 The Institute for the Development of Africa (The-IDA), Yaoundé, Cameroon,; 6 Laboratory of Virology, Hôpital Européen Georges Pompidou, and University of Paris Descartes, Paris Sorbonne Cité, Paris, France

**Keywords:** HIV, AIDS-related death, HIV-infected inpatients, predictors, Democratic Republic of the Congo

## Abstract

**Introduction:**

Human Immunodeficiency Virus (HIV) infection continues to be a major public health concern in sub-Saharan Africa. We aimed to evaluate potential factors associated with AIDS-related death among adult HIV-infected inpatients in Kisangani, the Democratic Republic of the Congo (DRC).

**Methods:**

this is a hospital-based retrospective, observational analysis carried out between 1^st^ January 2019 and 31^st^ March 2020 among inpatients HIV, at 12 facilities integrating the HIV prevention and care packages in Kisangani. Factors associated with AIDS-related death were analyzed using the logistic regression models.

**Results:**

a total of 347 HIV-infected inpatients were included. Among those, the rate of AIDS-related death was 25.1% (95% CI: 20.8-29.9). The rates of AIDS-related death were lower among patients with a university education (aOR: 0.03 [95% CI: 0.00-1.0]) and higher among patients in WHO clinical stage 4 (aOR: 15.4 [6.8-27.8]), patients with poor highly active antiretroviral therapy (HAART) observance (aOR: 14.5 [2.3-40.4), and patients suffering from opportunistic infections (aOR: 9.3 [95% CI: 3.4-25.1]), including cryptococcal meningitis (aOR: 27 [95% CI: 6.0-125.7]) and viral infections associated with zona and Kaposi sarcoma (aOR: 4.8 [95% CI: 2.2-10.4]).

**Conclusion:**

in our retrospective study on a large sample of inpatients hospitalized in Kisangani, classic causes of death were found. The association with the low level of education suggests that the economic level of the patients who die is a determining factor, difficult to correct. The identification of a limited number of other factors will allow a better medical management.

## Introduction

The human immunodeficiency virus (HIV) and its associated acquired immunodeficiency syndrome (AIDS) continue to be a major public health concern in sub-Saharan Africa, where nearly 25.7 million people are infected and approximately 70% of global deaths from HIV/AIDS occur [1, 2]. The wide availability of highly active antiretroviral therapy (HAART) since the mid-1990s has led to a significant reduction in AIDS-related hospitalizations and has improved the overall survival of those affected [3-5]. It has also reduced AIDS-definition rates caused by opportunistic infections and has altered the spectrum of HIV-related hospitalizations, which have become primarily due to chronic, non-AIDS-related conditions such as cardiovascular disease and malignancies [6]. Although several countries in Eastern and Southern Africa have achieved the 90-90-90 target [7], gaps persist in some Western and Central African countries [7], such as the Democratic Republic of the Congo (DRC), where the screening rate remains low (54%), resulting in delayed treatment and near-zero viral load-based biological monitoring [8-10]. Thus, in DRC, AIDS remains the main reason for hospitalization, with various opportunistic infections playing a major role in HIV-related morbidity and mortality [11].

Although several studies have been conducted in other sub-Saharan countries, the spectrum of HIV/AIDS-related diseases may present regional variations influenced by the local socio-economic, biological, health, and cultural context [12]. Better knowledge on AIDS in a given setting is therefore needed to guide policymakers and stakeholders to efficiently manage the resources available for the HIV response. It could also be important to raise awareness among health care workers about the issues most commonly encountered among HIV-positive patients to enable early diagnosis and effective management. Since published data on the causes of HIV-related hospitalizations, clinical profiles, and factors associated with the mortality of adult hospitalized patients with HIV in the DRC is scarce, we aimed to evaluate the potentials socio-economic and clinical factors associated with AIDS-related death among adult HIV-infected inpatients attending health facilities in Kisangani, the capital of the province of Tshopo, DRC.

## Methods

**Study design and setting:** this is a hospital-based retrospective, observational analysis carried out between 1^st^ January 2019 and 31^st^ March 2020 among inpatients HIV, at facilities integrating the HIV prevention and care packages in Kisangani. This survey was performed in all 12 facilities providing free care for people living with HIV in the capital city. At time of data collection, around 4,323 HIV-positive patients were followed-up with the following distribution: 642 patients in Makiso General Reference Hospital (GRH), 738 in Kabondo GRH, 385 in Lubunga GHR, 343 in Tshopo GHR, 360 in Mangobo GHR, 262 in Celpa medical center, 524 in Foyer reference health center (RHC), 484 in Mokili RHC, 129 in Saint Joseph RHC, 125 in Matete RHC, 84 in Alwaleed RHC, and 247 in University Hospital of Kisangani.

**Figure 1:**
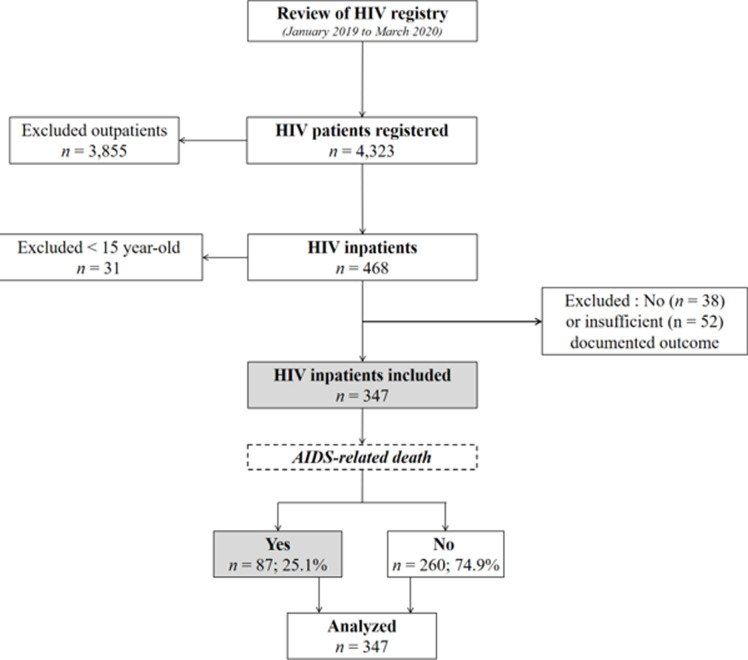
flow chart showing the review of HIV registry, inclusion of HIV inpatients, and rate of AIDS-related death

**Study population:** as depicted in Figure 1, study population were identified through the HIV testing and monitoring register in the 12 health care facilities. We included all HIV-positive inpatients over 15 years of age during our study period. All HIV-positive outpatients, HIV-positive inpatients with no outcome documented, and those with incomplete file information were excluded.

**Data collection:** the following variables were collected anonymously: demographic (age, sex, marital status, and educational level) and clinical (duration of HIV infection since diagnosis, number of known hospitalizations, duration of current hospitalization, clinical-stage according to WHO, HAART use and observance, access to viral load, cotrimoxazole and isoniazid prophylaxis use, diagnosis during hospitalization, and status upon discharge [alive or dead]). The four WHO clinical-stages was based on clinical evidence at the time of the last hospitalization [13]. The good observance was operationally defined as the regular HAART taking without interruption. Given the lack of CD4 testing for many HIV-infected hospitalized patients, the operational definition of AIDS-related disease was based on the WHO case definition of AIDS-related diseases that can occur in context of immunodepression and according the four clinical-stages that can be encountered in an HIV-positive individual [14]. Thus, the following clinical, biological, and imaging criteria were used to establish a diagnosis [12]: (i) pulmonary tuberculosis was incriminated in the event of a prolonged cough and fever, weight loss, positive sputum smear or culture for acid-fast bacilli or a compatible clinical presentation with suggestive findings on chest X-ray, a positive GeneXpert MTB/RIF (Cepheid, Sunnyvale, United States of America) result, and a response to anti-tuberculosis treatment; (ii) extrapulmonary tuberculosis was incriminated in the event of a prolonged fever, weight loss, histopathological diagnosis from an extrapulmonary site (e.g. lymph node; pleura) or a compatible clinical presentation and a response to anti-tuberculosis treatment; (iii) cerebral toxoplasmosis was incriminated in the event of a compatible clinical presentation (coma, hemiplegia, and other signs of localization), and a response to anti-toxoplasmosis treatment; (iv) cryptococcal meningitis was incriminated in the event of a compatible clinical presentation (intense headache, fever, coma, etc.) with the detection of cryptococcal antigen in cerebrospinal fluid or serum or the positivity in India ink; (v) candidiasis was incriminated in the event of a compatible clinical presentation (oral lily-of-the-valley, diarrhea, etc.) and positive culture in Sabouraud gelose; (vi) Coccidiosis, zona caused by varicella zona virus (VZV), and Kaposi syndrome associated with human herpesvirus 8 (HHV8) were essentially diagnosed on clinical basis (neurological focus and localization disorders for toxoplasmosis, chronic diarrhea with negative culture of *Candida albicans* in the Sabouraud gelose for coccidiosis [cryptosporidiosis, isosporosis, microsporosis or cyclosporosis], skin lesions for Kaposi and Zona); (vii) sepsis was diagnosed in the event of a systemic inflammatory response syndrome (SIRS) with an identified focus of infection (SIRS was defined as 2 or more of the following: fever > 38°C or < 36°C; heart rate > 90 beats per minute; respiratory rate > 20 breaths per minute or an arterial carbon dioxide tension < 32 mmHg; and white cell count > 12 000 or < 4000 cells/μL). The diagnosis of non-AIDS-related diseases was defined as any other disease that can occur even outside a context of immunodepression, and was made on the basis of clinical, biological, and imaging evidence.

**Statistical analysis:** we collected from the paper-based clinical records and contemporaneously entered it into an Excel database. The checks on data accuracy and plausibility were carried out by the facility data teams and the research team, including verifying a subset of database data against the original paper-based record. Finally, we exported data into SPSS 20.0 (Chicago, IL) for analyzes. First, descriptive statistics were computed using mean (standard deviation) or median (interquartile range) for normal or skewed distribution, respectively, then, the 95% confidence intervals (CI) using the Wilson score bounds were calculated. Next, categorical data were compared using Pearson´s Chi-square test, while Fisher´s exact test was used when the validity conditions of the latter test were not verified. Variables with a P-value < 0.05 in bivariate analysis and variables identified from the literature were entered into a full multivariate logistic regression model. The strength of statistical associations was measured by crude odds ratios (cOR) and adjusted odds ratios (aOR) and their 95% CI. All analyses were 2-sided and P-values of < 0.05 were considered statistically significant. Missing values that were less than 2% were replaced by the single imputation using regression method.

**Ethics statement:** the study was conducted after obtaining ethical approval from the Ethics Committee of the University of Kisangani. Furthermore, permission was obtained from provincial divisions of the health of the province of Tshopo, DRC. Anonymity was ensured by not gathering any personal information (name, telephone number, and residence) from the patients. Due to the nature of this study (retrospective collection of data on the basis of patient registers and follow-up sheets), obtaining informed consent was not required by the ethics committee.

## Results

A total of 4,323 HIV-positive patient records were reviewed, of which 3,976 were excluded because 3,855 concerned outpatients, 52 had incomplete file information, 38 had not outcome documented, and 31 patients were under 15 years of age. Finally, 347 HIV-positive inpatients records were included in this survey (Figure 1). Concerning the socio-demographic characteristics, more than half (53.9%) of HIV-positive inpatients were 40 years old or older (with a mean age of 40 years [SD: 13 years]); the majority of them were female (60.2%), married (77.2%), with low educational level (no formal education or completing primary school; 53.3%) (Table 1).

**Table 1:** socio-demographic and clinical characteristics of 347 inpatients infected with HIV in Kisangani, Democratic Republic of the Congo

Characteristics	Number or mean	Percent or SD
**Sociodemographic**		
**Age (years)**		
< 40	160	46.1
≥ 40	187	53.9
**Mean**	40	13
**Sex**		
Female	209	60.2
Male	138	39.8
**Partnership and civil status**		
Single	54	15.6
Married/partnered	268	77.2
Other	25	7.2
**Educational level**		
No formal education/ completing primary school	185	53.3
Attending college or technical school	102	29.4
University	60	17.3
**Clinical**		
**Duration of HIV infection since diagnosis, month (mean)**	14.6	12.5
**Number of known hospitalizations**		
1	269	77.5
2	59	17
≥ 3	19	5.5
**Duration of hospitalization, day (mean)**	10.1	9.2
**Clinical stage according to WHO**		
1	1	0.3
2	8	2.3
3	288	83
4	50	14.4
**Use of HAART**		
Yes	328	94.5
No	19	5.5
**Good observance of HAART**		
Yes	289	83.2
No	58	16.8
**Access to viral load**		
Yes	8	2.4
No	339	97.6
**Receiving cotrimoxazole prophylaxis**		
Yes	220	63.4
No	127	36.6
**Receiving isoniazid prophylaxis**		
Yes	7	2
No	340	98
AIDS-related diseases ^*^		
Yes	234	67.4
No	113	32.6
**Non-AIDS co-morbidity**		
Yes	49	14.1
No	298	85.9

* Among AIDS-related illnesses, opportunistic infections were found in 203 (86.6%) hospitalized patients. HAART: Highly Active Antiretroviral Therapy; SD: Standard Deviation; WHO: World Health Organization

**Figure 2:**
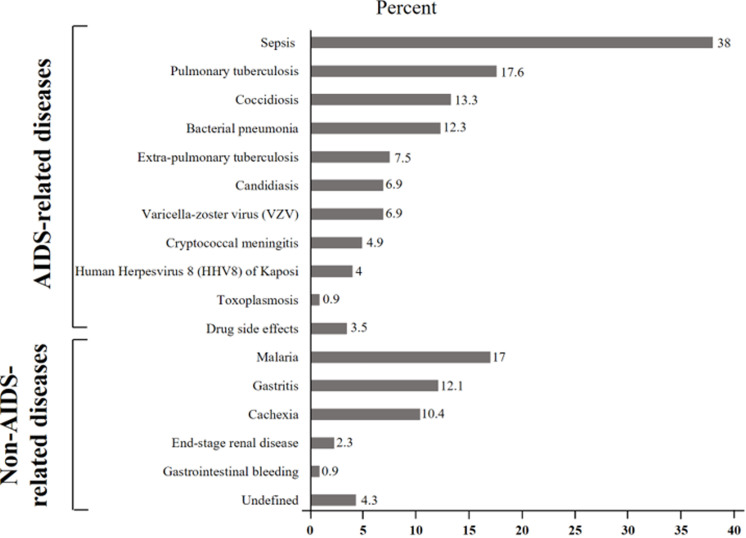
clustered bars showing the causes of hospitalization of HIV-infected patients in Kisangani, DRC

In terms of clinical features (Table 1), the mean duration of HIV infection from diagnosis to the time of the survey and the mean duration of hospital stay was 14.6 months (SD: 12.5 months) and 10.1 days (SD: 9.2 days), respectively. The vast majority of patients were at WHO stage 3 (83%) and were on antiretroviral therapy (94.5%). Less than one-fifth (16.8%) did not adhere well to antiretroviral therapy. Only 2.4% of the patients had received viral load testing. Although the majority (63.4%) received Cotrimoxazole prophylaxis, only 2% received Isoniazid prophylaxis. A total of 234 (67.4%) HIV-positive inpatients were diagnosed with AIDS-related illnesses; of these, opportunistic infections were found in 203 (86.6%) of cases. Sepsis (38%), pulmonary tuberculosis (17.6%), and coccidiosis (13.3%) were the AIDS-related illnesses frequently encountered in HIV-positive inpatients. While malaria (17%) and gastritis (12.1%) were the non-AIDS-related diseases frequently encountered in our series (Figure 2).

Overall, the rate of AIDS-related death was 25.1% (95% CI: 20.8-29.9). The risk factors associated with the AIDS-related death in our series are shown in Table 2. In bivariate analysis, the variables educational level, number of hospitalization, clinical stage according to WHO, use of HAART, good observance of HAART, and opportunistic infections were significantly associated with the AIDS-related death. Thus, the rate of AIDS-related death was higher among patients without formal education or completing primary school (33.5%), those hospitalized 3 times more (68.4%), those at WHO stage 4 (78%), those not using HAART (63.2%), those not correctly observing HAART (82.8%), and those with opportunistic infections (39.9%). However, in multivariate analysis, only the variables educational level, clinical stage according to WHO, good observance of HAART, and opportunistic infections remained associated with the AIDS-related death. Thus, the rates of AIDS-related death were lower among patients with a high education level (university) as compared to those with a low education level (aOR: 0.03 [95% CI: 0.00-1.0]), and higher among patients in WHO clinical stage 4 (aOR: 15.4 [6.8-27.8]), patients with poor HAART observance (aOR: 14.5 [2.3-40.4) and patients suffering from opportunistic infections (aOR: 9.3 [95% CI: 3.4-25.1]), including cryptococcal meningitis (aOR: 27 [95% CI: 6.0-125.7]) and viral infections associated with zona and Kaposi sarcoma (aOR: 4.8 [95% CI: 2.2-10.4]) (Table 3).

**Table 2:** socio-demographic and clinic characteristics associated with AIDS-related death among 347 inpatients infected with HIV in Kisangani, Democratic Republic of the Congo

Characteristic	AIDS-related death				
	Bivariate analysis		Multivariate analysis		
	Yes n (%)	cOR [95% CI]	P^*^	aOR [95% CI]	P^**^
**Educational level**					
No formal education/ completing primary school	62 (33.5)	1	< 0.001	1	1
Attending college or technical school	22 (21.6)	0.6 [0.3 - 0.9]		0.7 [0.3 - 1.1]	0.082
University	3 (5)	0.04 [0.01 - 1.3]		0.03 [0.00 - 1.0]	< 0.001
**Number of hospitalization**					
1	51 (19)	0.1 [0.04 - 0.3]	< 0.001	0.2 [0.04 - 0.7]	0.162
2	23 (39)	0.3 [0.1 - 0.9]		0.5 [0.1 - 2.5]	0.397
≥ 3	13 (68.4)	1		1	1
**Clinical stage according to WHO**					
3	46 (16)	1	< 0.001	1	1
4	39 (78)	18.7 [8.9 - 39.1]		15.4 [6.8 - 27.8]	0.038
**Use of HAART**					
Yes	75 (22.9)	0.2 [0.1 - 0.4]	< 0.001	0.3 [0.7 - 1.4]	0.137
No	12 (63.2)	1		1	1
**Good observance of HAART**					
Yes	39 (13.5)	1	< 0.001	1	1
No	48 ( 82.8)	30.8 [14.4 - 65.8]		14.5 [2.3 - 40.4	< 0.001
**Opportunistic infections**					
Yes	81 (39.9)	15 [6.4 - 36.2]	< 0.001	9.3 [3.4 - 25.1]	< 0.001
No	6 (4.2)	1		1	1

* P-value calculated using Pearson's χ2 test or Fisher's exact test;^**^ P-value calculated using logistic regression analysis. HAART: Highly Active Antiretroviral Therapy; WHO: World Health Organization.

**Table 3:** opportunistic infections associated with AIDS-related death among 347 inpatients infected with HIV in Kisangani, Democratic Republic of the Congo

Opportunist infections	AIDS-related death				
	Bivariate analysis		Multivariate analysis		
	Yes, n (%)	cOR [95% CI]	P^*^	aOR [95% CI]	P^**^
**Tuberculosis#**					
Yes	23 (24.4%)	1.1 [0.6 - 1.9]	0.734	-	
No	64 (24.6%)	1		-	
**Sepsis**					
Yes	21 (15.9%)	0.4 [0.2 - 0.7]	0.002	0.6 [0.3 - 1.1]	0.103
No	66 (30.7%)	1		1	1
**Candidiasis**					
Yes	0 (0%)	NA	0.003	-	-
No	87 (26.9%)	NA		-	-
**Cryptococcal meningitis**					
Yes	15 (88.2%)	6.9 [6.0 - 120.2]	< 0.001	27 [6.0 - 125.7]	< 0.001
No	72 (21.8%)	1		1	1
**Cerebral toxoplasmosis**					
Yes	2 (66.7%)	6.1 [0.5 - 68]	0.095	-	-
No	85 (27.7%)	1		-	-
**Coccidiosis**					
Yes	5 (10.9%)	0.3 [0.1 - 0.9]	0.017	0.7 [0.3 - 2.4]	0.124
No	82 (27.2%)	1		1	1
**Viral infections$**					
Yes	21 (58.3%)	5.2 [2.5 - 11]	< 0.001	4.8 [2.2 - 10.4]	< 0.001
No	66 (21.2%)	1		1	1

* P-value calculated using Pearson's χ2 test or Fisher's exact test; ^**^ P-value calculated using logistic regression analysis; # Of the hospitalized patients with tuberculosis who died, 16 (69.6%) had a smear-positive pulmonary, 5 (21.7%) had a smear-negative pulmonary, and 2 (8.7%) had extra-pulmonary tuberculosis; $ Among the viral infections related to death in this series, human herpes virus 8 (HHV8) infection associated with Kaposi's sarcoma was found in 14 (66.7%) inpatients; the varicella-zoster virus was found in seven (33.3%) inpatients

## Discussion

We herein examined retrospectively the factors and opportunistic infections associated with AIDS-related death during 15 months among adult HIV-infected inpatients in 12 health facilities which offer free testing and treatment to HIV-positive patients in the city of Kisangani, DRC. Our observations showed a high rate of AIDS-related death, around a quarter of HIV-infected inpatients. Low educational level, WHO clinical stage 4, poor HAART observance, and opportunistic infections were independently associated with the highest rates of AIDS-related deaths in our series. Among the opportunistic infections associated with AIDS-related death, cryptococcal meningitis and viral infections were the most frequent in multivariate analysis. Several cohort studies have demonstrated that HIV/AIDS-related conditions are the most frequent causes of death in hospitalized HIV-infected patients [15, 16]. In the present study, The HIV-related death rate among hospitalized HIV-positive patients was 25.1%. Although the 2017 report of the Joint United Nations Programme on HIV/AIDS (UNAIDS) estimates that the AIDS mortality rate has been declining since 2005 [17], a disproportionately high number of deaths (70%) have been recorded in sub-Saharan Africa, mainly in high-prevalence countries where the majority of people infected with HIV reside [17]. It should be noted that the proportion of deaths reported in the World Health Organization (WHO) region of West and Central Africa remains particularly striking. Although countries in this WHO region have consistently reported low HIV seroprevalence rates for many years [18], West and Central Africa accounted for 30% (280,000) of global mortality in 2017 [17], highlighting a regional public health problem of increasing scope and magnitude.

Previous studies in Nigeria [19], Ghana [20], Benin [21], Burkina Faso [21], Cote d'Ivoire [21], Mali [21], Senegal [21], and Sierra Leone [22] have similarly reported an AIDS-related death rate of around 30% among hospitalized patients, as in our study. Opportunistic infections are reported in Africa as the leading cause of hospitalization for AIDS-related deaths [12, 23, 24]. Similar findings were observed in our series. Although tuberculosis is not the leading cause of death in our series, it remains the most common presentation among HIV-infected people and is the leading cause of death among HIV-infected people worldwide according to the 2018 WHO report [13]. Although sepsis and tuberculosis were frequent causes of hospitalization of HIV-infected patients in our study, these illnesses were not statistically associated with death due to high-performance diagnostic tools (GeneXpert MTB/RIF, special culture media) for early diagnosis and treatments.

Cryptococcal meningitis and cancerous conditions of viral origin such as Kaposi's sarcoma were the most common opportunistic infections associated with AIDS-related death in our study. These conditions occur in HIV-infected patients whose CD4 count has collapsed (<100 cells/mm^3^). Although serum screening for cryptococcal antigens is reserved for an HIV-positive person having a CD4 T cell count of less than 100 cells/mm^3^ according to current WHO guidelines [25], a recent study highlights that a positive cryptococcal antigens result was strongly associated with an increased risk of cryptococcal meningitis or death [26]. Those authors suggest that raising the threshold for cryptococcal antigens screening to 200 cells/mm^3^ could help reduce morbidity and mortality in HIV-infected patients in cryptococcus-endemic regions [27]. Implementing a targeted screening of HIV patients with low body mass index, CD4 count < 100 cells, having headaches; and treatment for asymptomatic cryptococcal disease should be considered among inpatients infected by HIV in DRC. Additional data is needed to better define the epidemiology of cryptococcal antigenemia and its predictors in DRC in order to optimize the prevention, diagnosis, and treatment strategies [26].

The socio-demographic factor associated with death among the inpatients in this study was low educational level. This is justified by poor adherence to treatment among less-educated individuals [28]. The less educated individuals are often not in permanent employment and turn to informal activities to support their families. As a consequence, they are often victims of drug stock-outs and are often lost to long journeys away from their follow-up health facility. Intensive adherence counseling should be provided to all patients before the initiation of antiretroviral therapy. Health care providers must identify possible barriers to adherence at the earliest and provide appropriate solutions [28]. Furthermore, the fact that our study included HIV-infected patients who were not on HAART highlights another important gap in access to care services. Previous studies have shown that stigma, discrimination, fear of disclosure, the complexity of treatment regimens, lack of confidentiality, and lack of psychosocial support were factors that delayed diagnosis and initiation of treatment [29].

**Strengths and limitations:** the strength of this study is that it presents, to our knowledge, for the first time the factors associated with mortality among adults hospitalized with HIV/AIDS in DRC. However, there is some limitations in this study. Firstly, data about other potentially confounding biomedical predictors for death in our population, such as drug resistance, CD4 count, and viral load were not available and were thus excluded from analysis. Secondly, gaps in the accurate diagnosis of diseases lead to potential classification bias. Thirdly, the duration of known HIV infection is distinct from the duration of HIV infection and therefore underestimates the true duration of HIV infection. Finally, lack of CD4 testing in the immunological diagnosis of AIDS-related illnesses and deaths may constitute a classification bias. Furthermore, our study design did not allow us to follow HIV-infected patients over a long period to accomplish survival analysis, Cox model, and competing risks analysis method.

## Conclusion

This study showed that the AIDS-related death among inpatient in Kisangani, DRC is high. Low educational level, WHO clinical stage 4, poor HAART observance, and opportunistic infections (mainly cryptococcal meningitis and viral infections associated with zona and Kaposi sarcoma) were often associated with increased rates of AIDS-related deaths. In our retrospective study on a large sample of inpatients hospitalized in Kisangani, classic causes of death were found. The association with the low level of education suggests that the economic level of the patients who die is a determining factor, difficult to correct. The identification of a limited number of other factors will allow for better medical management.

### What is known about this topic

- The wide availability of HAART since the mid-1990s has led to a significant reduction in AIDS-related hospitalizations and mortality and has improved the overall survival of those affected;
- Various opportunistic infections playing a major role in HIV-related morbidity and mortality in DRC;
- Published data in the DRC on the causes of HIV-related hospitalizations, clinical profiles, and factors associated with the mortality of hospitalized patients with HIV are scarce.

### What this study adds

- This study showed that the rate of AIDS-related death among inpatient in Kisangani, DRC, was 25.1% (95% CI: 20.8 - 29.9);
- Low educational level, WHO clinical stage 4, poor HAART observance, and opportunistic infections were often associated with the increase in AIDS-related deaths.
